# Generation and manipulation of current-induced spin-orbit torques

**DOI:** 10.2183/pjab.97.025

**Published:** 2021-11-11

**Authors:** Kazuya ANDO

**Affiliations:** *1 Department of Applied Physics and Physico-Informatics, Keio University, Yokohama, Kanagawa, Japan.; *2 Keio Institute of Pure and Applied Science, Keio University, Yokohama, Kanagawa, Japan.; *3 Center for Spintronics Research Network, Keio University, Yokohama, Kanagawa, Japan.

**Keywords:** spintronics, spin-orbitronics, spin-orbit torques, spin Hall effect, Rashba effect

## Abstract

An emerging field of spintronics, spin-orbitronics, aims to discover novel phenomena and functionalities originating from spin-orbit coupling in solid-state devices. The development of spin-orbitronics promises a fundamental understanding of spin physics in condensed matter, as well as smaller, faster, and far-more energy-efficient spin-based devices. Of particular importance in this field is current-induced spin-orbit torques, which trigger magnetic dynamics by the transfer of angular momentum from an atomic lattice to local magnetization through the spin-orbit coupling. The spin-orbit torque has attracted extensive attention for its fascinating relativistic and quantum mechanical nature, as well as prospective nanoelectronic applications. In this article, we review our studies on the generation and manipulation of current-induced spin-orbit torques.

## 1 Introduction

For more than 200 years, the manipulation of magnetization has been a subject of human curiosity. In 1820, Oersted discovered that charge currents can manipulate nearby magnetic compass needles. This phenomenon is now understood in classical electromagnetism as that charge currents and magnetic moments are indirectly coupled by magnetic fields. In 1928, Dirac showed that electron spin is naturally derived by generalizing the Schrödinger equation to a relativistically covariant form.^1)^ Landau and Lifshitz proposed an equation of motion for magnetization based on phenomenological grounds in 1935.^2)^ In 1955, Gilbert modified this equation by introducing a more convincing form for the damping term.^3)^ The combined form is now called the Landau-Lifshitz-Gilbert (LLG) equation, which has proven to be an indispensable and versatile tool to describe a wide range of magnetic and spintronic phenomena. The Dirac equation also predicts a relativistic interaction of an electron’s spin with its momentum: spin-orbit coupling. The spin-orbit coupling in systems with broken inversion symmetry was described in 1960, which is known as the Bychkov-Rashba spin-orbit coupling.^4)^ In 1971, Dyakonov predicted the spin Hall effect, a phenomenon that converts a charge current into a spin current through the spin-orbit coupling.^5)^ The Bychkov-Rashba spin-orbit coupling and spin Hall effect are now central in spintronics.

In 1996, a breakthrough took place in the manipulation of magnetization; Slonczewski and Berger independently predicted that a charge current can manipulate the magnetization of a magnet through a torque, called a spin-transfer torque, arising from direct action of the current spin polarization on local magnetic moments.^6,7)^ Ever since this prediction, intensive efforts have been devoted to incorporate the spin degree of freedom into charge-based electronic devices, and the manipulation of magnetization by the current-induced spin torques turns out to be a key ingredient of spintronics.

The recent discovery of a new type of spin torques, originating from spin-orbit coupling, has led to another breakthrough in the field of spintronics.^8)^ In solids, the spin-orbit coupling triggers the transfer of orbital angular momentum from the lattice to the spin system, which results in exerting a toque on the local magnetic moment. The current-induced torque arising from the spin-orbit coupling is called spin-orbit torques, which provide an efficient and versatile way to control the magnetic state and dynamics in various classes of materials, such as heterostructures without inversion-symmetry^9–11)^ and noncentrosymmetric magnets.^12,13)^ This progress has led to an emerging direction in spintronics that aims at discovering novel phenomena and functionalities originating from spin-orbit coupling, called spin-orbitronics.^14–34)^

Here, we review our studies on the generation and manipulation of current-induced spin-orbit torques. This review is organized as follows. In Sec. 2, we introduce the concept of spin-orbit torques. Section 3 describes the spin-torque ferromagnetic resonance, a method for measuring the spin-orbit torques. Section 4 describes the spin-orbit torques in metallic devices, the prototypical spin-orbitronic device. In Secs. 5–9, we discuss our recent studies on spin-orbit torques. Conclusions are given in Sec. 10.

## 2 Spin-orbit torques

**Figure 1.**
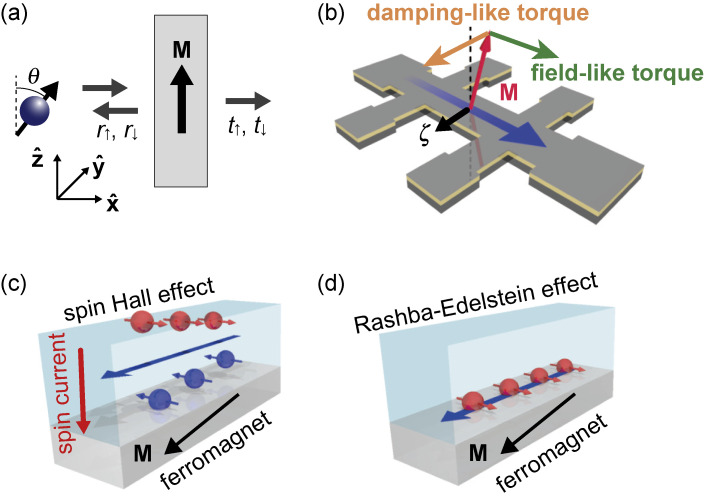
(Color online) (a) Injection of an electron into a thin ferromagnetic layer with its magnetization along $\hat{z}$ . The transmission, *t*_↑(↓)_, and reflection, *r*_↑(↓)_, amplitudes are dependent on spin ↑(↓). (b) The spin torques: the damping-like (DL) torque (orange arrow) and the field-like torque (green arrow). (c) Conversion from a charge current into a transverse spin current by the spin Hall effect. (d) Nonequlibrium in-plane spin polarization developed by the Rashba-Edelstein effect. Figures adapted from Ref. 37.

We first describe an elementary model of current-induced spin torques.^35–37)^ The behavior of a spin current at nonmagnetic/ferromagnetic interfaces is dominated by spin-dependent reflection and transmission of electrons. Figure 1(a) illustrates a situation where a single electron is incident onto a thin ferromagnetic film whose magnetization, **M**, is directed in the $\hat{z}$ direction. The wavevector **k** of the incident electron is in the $\hat{x}$ direction and the spin direction is in the $\hat{x}$ – $\hat{y}$ plane at an angle θ with respect to the $\hat{z}$ direction. The wavefunction of the incident electron is expressed as

$$\psi_{\text{in}}=\frac{e^{ikx}}{\sqrt{\Omega }}(\cos(\theta /2)|↑\rangle +\sin(\theta /2)|↓\rangle ).$$ [1]

In the nonmagnetic/ferromagnetic junction, because of the exchange interaction in the ferromagnetic layer, the reflection and transmission amplitudes for the electron depend on the electron’s spin. By taking into account the spin-dependent transmission, *t*_↑(↓)_, and reflection, *r*_↑(↓)_, amplitudes for the incident electron with the spin ↑(↓), the transmitted and reflected parts of the scattering wavefunction in the absence of spin-flipping processes are expressed as

$$\psi_{\text{trans}}=\frac{e^{ikx}}{\sqrt{\Omega }}(t_{↑}\cos(\theta /2)|↑\rangle +t_{↓}\sin(\theta /2)|↓\rangle ),$$ [2]

$$\psi_{\text{refl}}=\frac{e^{-ikx}}{\sqrt{\Omega }}(r_{↑}\cos(\theta /2)|↑\rangle +r_{↓}\sin(\theta /2)|↓\rangle ).$$ [3]

For ψ_in_, ψ_trans_, and ψ_refl_, the incident **Q**_in_, transmitted **Q**_trans_, and reflected **Q**_refl_ components of the spin current density flowing in the $\hat{x}$ direction can be calculated using

$$Q=\frac{\hbar^{2}}{2m}\text{Im}(\psi^{*}\sigma ⊗\nabla \psi ),$$ [4]

which is analogous to the probability current density, given by (*ħ*/*m*) Im(ψ*∇ψ). Here, ψ is a single-electron wavefunction, *m* is the electron mass, and $\sigma =(\sigma_{x},\sigma_{y},\sigma_{z})$ represents the Pauli matrices. The spin current density is a tensor quantity because it has both a direction in spin space and a direction of flow in real space.

The spin current density flowing left of the ferromagnetic layer, **Q**_in_ + **Q**_refl_, is not equal to the spin current density on the right **Q**_trans_. Because of the conservation of angular momentum, the lost spin component is transferred to the ferromagnetic layer in the form of a torque, which is the spin torque.^35)^ The net spin current transferred from the electron to the ferromagnetic layer on the area *A* is equal to the spin torque **N**_st_ on the area: $N_{\text{st}}=A\hat{x}\cdot (Q_{\text{in}}+Q_{\text{refl}}-Q_{\text{trans}})$ , where **N**_st_ is a vector in spin space. Using |*t*_↑_|^2^ + |*r*_↑_|^2^ = 1 and |*t*_↓_|^2^ + |*r*_↓_|^2^ = 1, the spin torque is expressed as

$$\begin{array}{rl} N_{\text{st}} & =\frac{A}{\Omega }\frac{\hbar^{2}k}{2m}\sin\theta [1-\text{Re}(t_{↑}t_{↓}^{*}+r_{↑}r_{↓}^{*})]\hat{x}\\ & \;-\frac{A}{\Omega }\frac{\hbar^{2}k}{2m}\sin\theta \text{Im}(t_{↑}t_{↓}^{*}+r_{↑}r_{↓}^{*})\hat{y}. \end{array}$$ [5]

Equation [5] shows that non-zero torque perpendicular to the magnetization is induced when the incident spin orientation is non-collinear with the magnetization (θ ≠ 0) and spin filtering is present (*t*_↑_ ≠ *t*_↓_, *r*_↑_ ≠ *r*_↓_). The Landau-Lifshitz-Gilbert equation, by taking into account the spin torques, is expressed as^8)^

$$\begin{array}{rl} \frac{dM}{dt} & =-\gamma M\times H_{\text{eff}}+\frac{\alpha }{M_{\text{s}}}M\times \frac{dM}{dt}\\ & \;+\tau_{\text{FL}}M\times \zeta +\tau_{\text{DL}}M\times (M\times \zeta ), \end{array}$$ [6]

since $\hat{x}∥M\times (M\times \zeta )$ and $\hat{y}∥M\times \zeta$ . Here, γ is the gyromagnetic ratio, **M** is the magnetization in the ferromagnet, α is the magnetic damping, *M*_s_ is the saturation magnetization, **ζ** is the unit vector along the incident spin orientation, **H**_eff_ is the effective magnetic field, including such as the applied magnetic fields and demagnetization fields. The torque with the form of τ_DL_**M** × (**M** × **ζ**) acts like an effective magnetic damping, and is called a damping-like torque. In contrast, the torque with the form of τ_FL_**M** × **ζ** acts on the magnetization like an effective magnetic field, and is referred to as a field-like torque. Figure 1(b) shows the directions of these two torque components.

In realistic three-dimensional nonmagnetic/magnetic heterostructures, the calculation requires summing the torque, Eq. [5], throughout the Fermi surface of the nonmagnetic layer. The resulting field-like torque, τ_FL_, and damping-like torque, τ_DL_, can be expressed in terms of the spin mixing conductance,^38)^

$$G_{↑↓}=\frac{e^{2}}{h}\sum_{n\in \text{NM}}\left(1-\sum_{m\in \text{NM}}r_{↑}r_{↓}^{*}\right),$$ [7]

where *n* and *m* are the transport channel index on the nonmagnetic side of the contact. When the magnetic layer is thick enough to neglect *t*_↑_ and *t*_↓_, the damping-like and field-like torques can be simplified as $\tau_{\text{DL}}∼\text{Re}(1-r_{↑}r_{↓}^{*})$ and $\tau_{\text{FL}}∼\text{Im}(r_{↑}r_{↓}^{*})$ . Thus, the damping-like torque corresponds to the real part of *G*_↑↓_, τ_DL_ ∼ Re[*G*_↑↓_], while the field-like toque corresponds to the imaginary part of *G*_↑↓_, τ_FL_ ∼ Im[*G*_↑↓_].

The elementary model predicts that a nonzero spin polarization in the nonmagnetic layer can give rise to the spin torques. The spin torques originating from the transfer of orbital angular momentum from the lattice to the spin system triggered by spin-orbit coupling are the current-induced spin-orbit torques. The spin-orbit coupling is the relativistic interaction between electrons’ spin and momentum and is central to magnetism and spintronics.^8,39–43)^ Of particular recent interest is the spin-orbit coupling in heterostructures with broken inversion-symmetry, such as heavy-metal/ferromagnetic-metal bilayers. The spin-orbit coupling in such systems gives rise to several microscopic mechanisms of the current-induced spin-orbit torques.^8)^

In one picture, the bulk spin-orbit coupling in the heavy metal layer is responsible for the generation of spin-orbit torques. In the heavy metal, the bulk spin-orbit coupling converts an applied charge current into a transverse spin current, which is known as the spin Hall effect (SHE).^9,41–52)^ The generated spin current is subsequently absorbed in the adjacent ferromagnetic layer in the heavy-metal/ferromagnetic-metal bilayer (see also Fig. 1(c)); the angular momentum carried by the spin Hall current is transferred to the magnetization, inducing the spin-orbit torques.

The spin-orbit coupling at the heavy-metal/ferromagnetic-metal interface also plays an essential role in the generation of the damping-like and field-like torques. At the interface, the inversion symmetry is broken along the normal to the interface, $\hat{z}$ . The inversion symmetry breaking modifies the orbital hybridization close to the nucleus, where the spin-orbit coupling is strong. The symmetry-breaking-induced modification of the orbital hybridization near the interface, in conjunction with the spin-orbit interaction, results in the so-called Rashba spin-orbit coupling^4)^: $H_{\text{R}}=\alpha_{\text{R}}\sigma \cdot (\hat{z}\times k)$ , where α_R_ is the Rashba parameter, **σ** is the vector of the Pauli spin matrices, and **k** is the electron wavevector. The Rashba spin-orbit coupling lifts the electron-spin degeneracy, and the spin angular momentum is locked on the linear momentum.^53)^ Because of the spin-momentum locking, a nonzero spin accumulation is generated by applying an electric field parallel to the interface, a phenomenon called the Rashba-Edelstein or inverse spin galvanic effect (see also Fig. 1(d)).^54,55)^ Since the Rashba spin-orbit effective field induces rotation of the spin accumulation, both field-like and damping-like torques can be generated through the exchange coupling at the heavy-metal/ferromagnetic-metal interface.^10,13,34,56–61)^

## 3 Spin-torque ferromagnetic resonance

The current-induced spin-orbit torques change the magnetization angle relative to the external magnetic field due to the activation of resonant and nonresonant oscillations, coherent rotation, or domain wall motion. These dynamic changes are detectable by either electrical or optical method. This is the foundation of multiple different techniques developed to quantify the spin-orbit torques.^11,12,16,62–67)^ Among them, the spin-torque ferromagnetic resonance (ST-FMR) is widely used to quantify the spin-orbit torques in various systems.^11,12)^

For the ST-FMR measurement for a nonmagnetic/ferromagnetic bilayer, we apply a radio frequency (RF) current of *I*(*t*) = *I* cos(ω*t*). In the device, the spin-orbit torques, as well as an Oersted field, due to the RF current induce the oscillation of magnetization in the ferromagnetic layer under the FMR condition. This results in time-dependent resistance, *R*(*t*) = *R*_∥_ − Δ*R* cos^2^ θ(*t*), due to anisotropic magnetoresistance (AMR), where *R*_∥_ is the resistance when the current is parallel to the magnetization and Δ*R* is the resistance change due to the AMR. Here, θ(*t*) = θ + θ_c_ cos(ω*t* − ψ), where θ is the angle between the external field, *H*, and the applied RF current, θ_c_ is the cone angle of the magnetization precession, and ψ is the phase difference. For small θ_c_, by expanding the cos^2^ θ(*t*) term to the first-order approximation, the voltage across the device, *V*(*t*) = *I*(*t*)*R*(*t*), has a time-independent voltage: *V*_mix_ = (1/2)Δ*RI*θ_c_ cos ψ sin 2θ, which is the ST-FMR signal.

For thin film systems with negligible in-plane magnetic anisotropy, θ_c_ can be obtained from Eq. [6] by taking into account the damping-like and field-like effective fields, as well as the Oersted field, the external magnetic field, and the demagnetization field. Owing to the strong demagnetization field, the trajectory of the magnetization precession is highly elliptical with the semi-major axis lying in the film plane. When the precession angle is small, *V*_mix_ is dominated by the in-plane cone angle of the magnetization precession, θ_c_ ≈ *m*_*y*__′_/*M*_s_, where *m*_*y*__′_ is the in-plane magnetization-precession amplitude. By solving Eq. [6] with the above assumption, the direct-current (DC) voltage induced by the ST-FMR is obtained as^11)^:

$$\begin{array}{rl} V_{\text{mix}} & =S\frac{W^{2}}{(\mu_{0}H-\mu_{0}H_{\text{FMR}}{)}^{2}+W^{2}}\\ & \;+A\frac{W(\mu_{0}H-\mu_{0}H_{\text{FMR}})}{(\mu_{0}H-\mu_{0}H_{\text{FMR}}{)}^{2}+W^{2}}, \end{array}$$ [8]

where *S* and *A* are the magnitude of the symmetric and antisymmetric components, respectively. *W* = αω/γ is the spectral width and *H*_FMR_ is the FMR field. The symmetric part is proportional to the damping-like effective field, *H*_DL_, and the antisymmetric part is due to the sum of the Oersted field, *H*_Oe_, and the field-like effective field, *H*_FL_ as^68)^:

$$\begin{array}{rl} S & =\frac{I\Delta R}{2}\mu_{0}H_{\text{DL}}\frac{\mu_{0}H_{\text{FMR}}+\mu_{0}M_{\text{eff}}}{W(2\mu_{0}H_{\text{FMR}}+\mu_{0}M_{\text{eff}})}\\ & \;\times \sqrt{\frac{\mu_{0}H_{\text{FMR}}}{\mu_{0}H_{\text{FMR}}+\mu_{0}M_{\text{eff}}}}\sin2\theta \cos\theta , \end{array}$$ [9]

$$\begin{array}{rl} A & =\frac{I\Delta R}{2}(\mu_{0}H_{\text{FL}}+\mu_{0}H_{\text{Oe}})\\ & \;\times \frac{\mu_{0}H_{\text{FMR}}+\mu_{0}M_{\text{eff}}}{W(2\mu_{0}H_{\text{FMR}}+\mu_{0}M_{\text{eff}})}\sin2\theta \cos\theta , \end{array}$$ [10]

where ψ is assumed to be negligibly small. *M*_eff_ is the effective demagnetization field.

To characterize the spin-orbit torques in nonmagnetic/ferromagnetic bilayers, we define the damping-like (DL) and field-like (FL) torque efficiencies as

$$\xi_{\text{DL(FL)}}=\frac{2e}{\hbar }\mu_{0}M_{\text{s}}d_{\text{FM}}\frac{H_{\text{DL(FL)}}}{j_{\text{c}}},$$ [11]

where *d*_FM_ is the ferromagnetic-layer thickness and *j*_c_ is the charge current density in the nonmagnetic layer. The spin-orbit torque efficiencies can be determined using Eq. [11] from the magnitude of *S* and *A* with the RF current density, *j*_c_, flowing in the nonmagnetic layer. For a nonmagnetic/ferromagnetic bilayer, *j*_c_ can be estimated by monitoring the current-induced resistance change due to Joule heating.^69)^ However, this method is hard to apply to some systems because it requires one to determine the RF-current distribution in the device. Another method is to use the Oersted field to estimate the current density in the nonmagnetic layer. In the bilayer, the Oersted field acting on the magnetization is expressed as *H*_Oe_ = *j*_c_*d*_N_/2, where *d*_N_ is the thickness of the nonmagnetic layer. Using this relation with Eqs. [9]–[11], we obtain^68)^

$$\frac{1}{\xi_{\text{FMR}}}=\frac{1}{\xi_{\text{DL}}}\left(1+\frac{\hbar }{e}\frac{\xi_{\text{FL}}}{\mu_{0}M_{\text{s}}d_{\text{FM}}d_{\text{N}}}\right),$$ [12]

where

$$\xi_{\text{FMR}}=\frac{S}{A}\frac{e\mu_{0}M_{\text{s}}d_{\text{FM}}d_{\text{N}}}{\hbar }\sqrt{1+\frac{\mu_{0}M_{\text{eff}}}{\mu_{0}H_{\text{FMR}}}}$$ [13]

is the FMR spin-torque generation efficiency. Equation [12] indicates that the spin-orbit torque efficiencies, ξ_DL(FL)_, can be determined by measuring *d*_F_ dependence of ξ_FMR_. Here, notable is that ξ_FMR_ is determined by the spectral shape, or *S*/*A*, and this method does not require calculating the RF-current distribution in the device; this method is self-calibrated in the sense that the spin-orbit effective fields are measured relative to the Oersted filed. Similarly, the damping-like and field-like torque efficiencies per applied electric field *E*, defined as $\xi_{\text{DL}(\text{FL})}^{E}=(2e/\hbar )\mu_{0}M_{\text{s}}d_{\text{FM}}H_{\text{DL}(\text{FL})}/E$ , can be determined using

$$\frac{1}{\xi_{\text{FMR}}}=\frac{1}{\xi_{\text{DL}}^{E}}\left(\frac{1}{\rho_{\text{N}}}+\frac{\hbar }{e}\frac{\xi_{\text{FL}}^{E}}{\mu_{0}M_{\text{s}}d_{\text{FM}}d_{\text{N}}}\right),$$ [14]

where ρ_N_ is the resistivity of the nonmagnetic layer.

Under the FMR, the precession of the magnetization drives spin pumping, which injects a spin current into the nonmagnetic layer.^70)^ The injected spin current is converted into an electric voltage through the inverse spin Hall effect and/or the inverse Rashba-Edelstein effect.^15,51,71)^ The spin pumping also contributes to the symmetric voltage in the ST-FMR spectra.^72,73)^ The contribution from the spin pumping to the observed symmetric voltage is non-negligible only in devices with a small AMR of the ferromagnetic layer because the AMR is the source of ST-FMR signals, while the spin-pumping contribution is irrelevant to the AMR. The spin-pumping contribution in ξ_FMR_ decreases with increasing the frequency of the RF current, while the ST-FMR predicts that ξ_FMR_ is independent of the frequency, showing that the frequency dependence of ξ_FMR_ provides information on the contribution from the spin pumping to the measured signals. Here, the spin-pumping contribution can be neglected in devices having ferromagnetic layers with a large AMR, such as Ni_81_Fe_19_.^24,72,73)^

## 4 Spin-orbit torques in metallic devices

**Figure 2.**
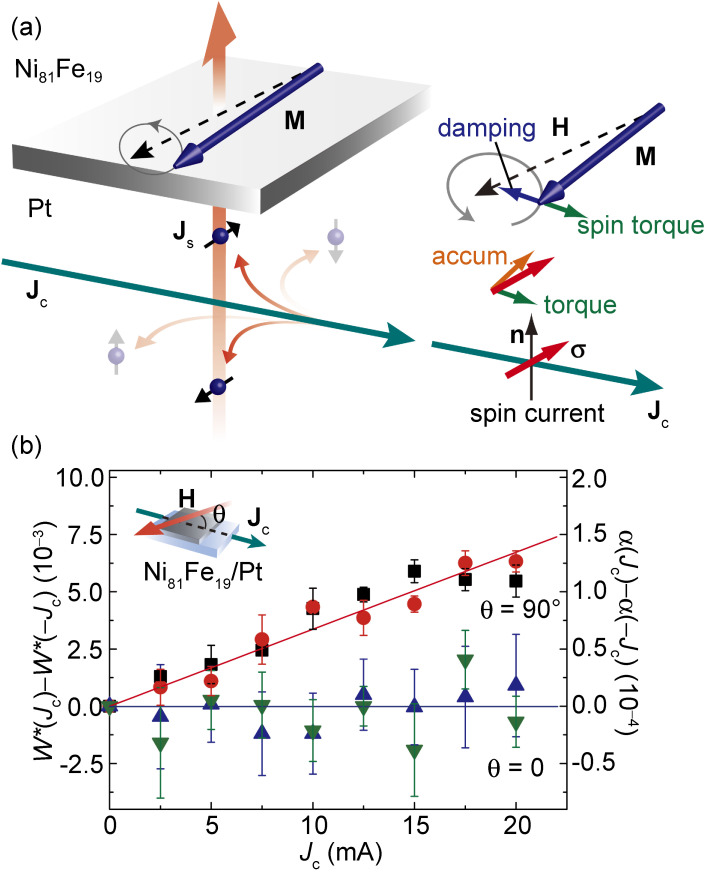
(Color online) (a) Schematic illustration of the spin-Hall and the spin-torque effects in the Pt/Ni_81_Fe_19_ film. **M**, **J**_s_, and **σ** denote the magnetization in the Ni_81_Fe_19_ layer, the flow direction of the spin current, and the spin polarization vector of the spin current, respectively. (b) Applied charge current, *J*_c_, dependence of Δ*W* ≡ W*(*J*_c_) − W*(−*J*_c_) for the Ni_81_Fe_19_/Pt bilayer film at θ = 90° (black and red) and 0 (blue and green). Here, W*(*J*_c_) ≡ *W*(*J*_c_)/*W*(0), where *W*(0) is the FMR linewidth at *J*_c_ = 0. The *J*_c_ dependence of Δα ≡ α(*J*_c_) − α(−*J*_c_) is also plotted, where α represents the magnetic damping constant. The data in different colors, namely, the red (the blue) and the black (the green) data, were measured in different samples. The lines in red and blue are linear fits to the data at θ = 90° and Δα = 0, respectively. Figures adapted from Ref. 9.

The initial work on the spin-orbit torques demonstrated the ability of the spin Hall effect to modify the dynamics of the magnetization in a Pt/Ni_81_Fe_19_ bilayer.^9)^ Figure 2(a) shows a schematic illustration of the spin-Hall and the spin-torque effects in the Pt/Ni_81_Fe_19_ film. For the Pt/Ni_81_Fe_19_ film, ferromagnetic resonance (FMR) was measured with applying a charge current, **J**_c_. In the Pt layer, the spin Hall effect converts **J**_c_ into a spin current, **J**_s_. The spin current is injected into the Ni_81_Fe_19_ layer through the interface, as shown in Fig. 2(a). From this measurement, we found that the FMR spectral shape is modulated by the applied charge current. Notable is that the FMR linewidth, *W*, is changed by the applied charge current only when the magnetic field is applied perpendicular to the charge current, as shown in Fig. 2(b). This result demonstrates that the magnetic damping constant, α, is manipulated by the applied charge current, since α is proportional to *W*.

The observed manipulation of the magnetic damping is caused by spin transfer induced by the spin Hall effect. We found that the current-induced damping modulation is absent in Ni_81_Fe_19_/Cu and Ni_81_Fe_19_ films, showing that the strong spin-orbit coupling in the Pt layer is responsible for the current-induced damping modulation. In the Pt/Ni_81_Fe_19_ bilayer, the damping-like torque due to the spin Hall effect draws the magnetization in the Ni_81_Fe_19_ layer toward or away from the external magnetic field direction, depending on the current direction. Since this torque is parallel or anti-parallel to the Gilbert-damping torque, it modulates the magnetic damping constant. Following this study, the spin Hall effect in Pt has been shown to drive the FMR by an alternating in-plane current in the Pt/Ni_81_Fe_19_ film.^11)^ The current-induced damping modulation and ST-FMR are now widely used to quantify the strength of the current-induced spin-orbit torques in a variety of materials.

Understanding the physics behind generating the spin-orbit torques is essential for a fundamental understanding of spin-dependent transport in solid-state devices. Since the first observation of the spin-orbit torque more than a decade ago, Pt has been central for establishing the physics of the spin-orbit torque.^9,10,74)^ A wide range of experiments have demonstrated that the spin-orbit torques can be manipulated by materials and interface engineering in Pt-based structures.^31,75–79)^ However, despite significant progress, the origin of the spin-orbit torques is still controversial, even in the prototypical spin-orbitronic device. An example is the spin-orbit torque in Pt/Ni-Fe-alloy bilayers, where the magnitude and sign of the field-like torque are inconsistent in literature.^31,76,80,81)^ We found that, by investigating the ST-FMR for Pt/ferromagnetic-metal bilayers, the ferromagnetic layer, as well as the heavy metal layer, plays an important role in generating the spin-orbit torque.^82)^

**Figure 3.**
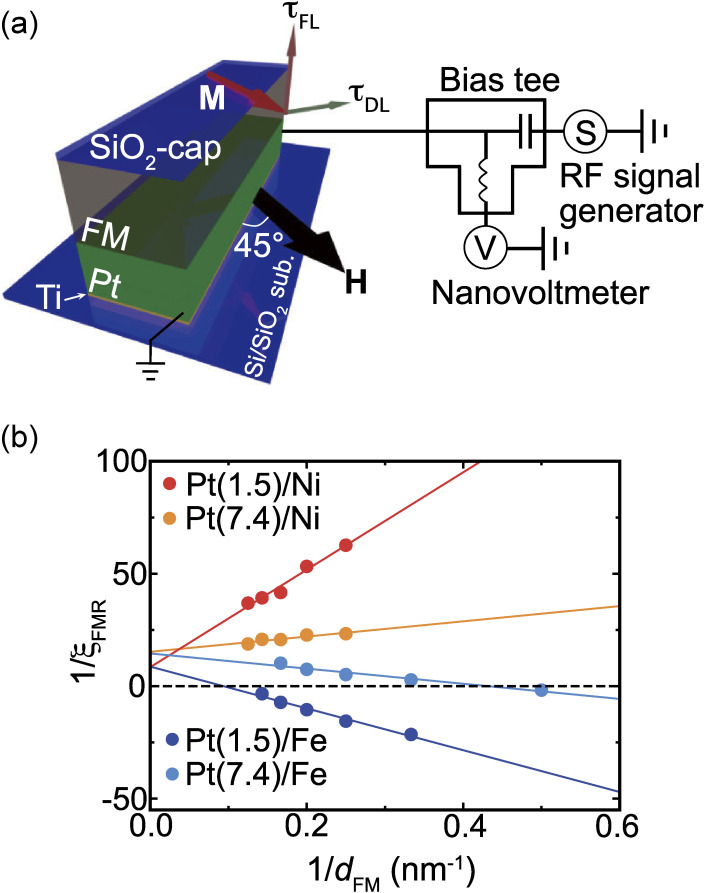
(Color online) (a) Schematic illustration of the experimental set up of the ST-FMR measurement for Pt/FM (FM = Ni and Fe) bilayers. (b) The 1/*d*_FM_ dependence of 1/ξ_FMR_ for the Pt/Ni and Pt/Fe bilayers, where *d*_FM_ is the thickness of the ferromagnetic layer and ξ_FMR_ is the FMR spin-torque efficiency at *f* = 9 GHz for the Pt/Ni bilayer and *f* = 16 GHz for the Pt/Fe bilayer. The numbers in parentheses represent the thickness in units of nanometers. The solid circles are the experimental data and the solid lines are the linear fitting result. Figures adapted from Ref. 82.

Figure 3(a) shows a schematic illustration of the experimental setup of the ST-FMR measurement for the Pt/FM (FM = Ni and Fe) bilayers with a size of 10 µm × 150 µm. For ST-FMR measurements, an RF charge current is applied along the longitudinal direction of the device and an in-plane external magnetic field *H* is applied with an angle of 45° from the longitudinal direction. The RF current generates damping-like and field-like torques, as well as an Oersted field, driving magnetization precession in the FM layer.^68)^ The magnetization precession induces an oscillation of the bilayer resistance due to the anisotropic magnetoresistance; the DC voltage, *V*_mix_, is generated through mixing of the RF charge current and oscillating resistance (see Eq. [8]).

Figure 3(b) shows the 1/*d*_FM_ dependence of 1/ξ_FMR_ for the Pt/FM (FM = Ni and Fe) bilayers. This result shows that the sign of the intercept of the linear relation is positive in all devices, showing $\xi_{\text{DL}}^{E}>0$ in the Pt/Ni and Pt/Fe bilayers. In contrast, the opposite sign of the slope between the Pt/Ni and Pt/Fe bilayers indicates that the sign of $\xi_{\text{FL}}^{E}$ is opposite between the Pt/Ni and Pt/Fe bilayers: $\xi_{\text{FL}}^{E}>0$ in the Pt/Ni film and $\xi_{\text{FL}}^{E}<0$ in the Pt/Fe film. We found that the magnitude of $\xi_{\text{DL}}^{E}$ is almost identical in the Pt/Ni and Pt/Fe bilayers, despite the stronger spin memory loss at the Pt/Fe interface. This suggests that the Pt/Fe interface, as well as the Pt bulk, contributes to the damping-like torque in the Pt/Fe bilayer. The ST-FMR result also shows that the sign of the field-like torque efficiency, $\xi_{\text{FL}}^{E}$ , which depends on the Pt-layer thickness, was found to be opposite between the Pt/Ni and Pt/Fe bilayers, showing that the direction of the field-like torque due to the bulk spin Hall effect is opposite between the Pt/Ni and Pt/Fe bilayers. This can be attributed to the opposite sign of the imaginary part of the spin-mixing conductance; the sign of the imaginary part of the spin mixing conductance, which is due to spin-dependent reflection arising from a spin-dependent potential at the interface, can be different depending on the choice of the ferromagnetic layer.^83,84)^ The clear difference in the spin-orbit torques in the Pt/FM bilayers shows that the electronic structure of the ferromagnetic layer, as well as the interfacial spin-orbit coupling, plays an important role in generating the spin-orbit torques.

An efficient way to manipulate the spin-orbit torque originating from the spin Hall effect is alloying. By changing the combination of the host and impurity metals or by changing the concentration of the impurities,^85,86)^ the engineering of the spin Hall effect has been reported for a variety of systems, including Cu, Pt, and Au-based alloys.^20,22,23,77,87–97)^ We demonstrated that the spin Hall effect in Au_100−__*x*_Cu_*x*_ changes drastically with the Cu concentration, *x*.^22)^ By changing the Cu concentration, we found that the sign of the effective spin Hall angle becomes negative only when 5 < *x* < 16, despite the positive spin Hall angle of pure Au and Cu. The sign reversal can be attributed to the spin Hall effect due to skew scattering in Au with dilute Cu impurities. Furthermore, we observed the crossover of the spin Hall effect between the two distinct regimes, the extrinsic impurity scattering and the intrinsic Berry curvature mechanism, induced by tuning the composition of the Au-Cu alloy. The tunable spin Hall effect enables one to control the magnitude and sign of the spin-torque in a Au_1−__*x*_Cu_*x*_/Ni_81_Fe_19_ structure. In this system, the spin-orbit torque efficiency is maximized at around *x* = 50, where the electric resistivity is also maximized due to the maximum atomic disorder scattering.

## 5 Spin-orbit torque engineering by oxygen manipulation

**Figure 4.**
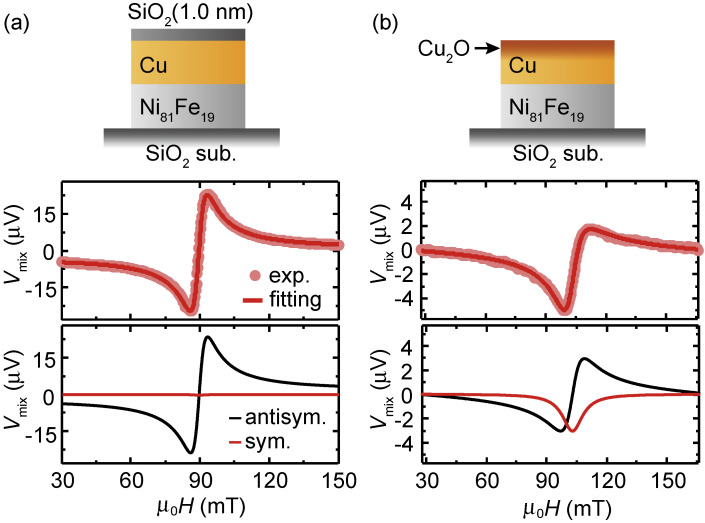
(Color online) Magnetic field, μ_0_*H*, dependence of the DC voltage, *V*_mix_, for (a) the SiO_2_(1 nm)/Cu(10 nm)/Ni_81_Fe_19_(8 nm) device and (b) the Cu(10 nm)/Ni_81_Fe_19_(8 nm) device at 7 GHz. The solid circles are the experimental data and the solid curves are the fitting result. The symmetric and antisymmetric components of the fitting results are plotted correspondingly. Figures adapted from Ref. 30.

The spin-orbit torques in metallic devices can be engineered by oxygen manipulation.^28–32,79)^ The most dramatic effect induced by the oxidation was demonstrated in the generation of a spin-orbit torque by Cu.^30)^ Cu is an archetypal metal with weak spin-orbit coupling, whose spin Hall angle is two orders of magnitude lower than that of Pt. Figure 4(a) shows the ST-FMR spectra for a Cu/Ni_81_Fe_19_ bilayer, capped by a SiO_2_ layer. The result shows that the ST-FMR signal is almost antisymmetric and the symmetric component is negligibly small, indicating a negligible damping-like torque in this system. The negligible damping-like torque is consistent with the prediction of weak spin-orbit coupling and a weak spin Hall effect in Cu. We found that the damping-like torque can be tuned by controlling the surface oxidization. Figure 4(b) shows the ST-FMR spectra for a Cu/Ni_81_Fe_19_ bilayer where the surface of the Cu layer is oxidized by exposing the surface to the laboratory ambient. This result demonstrates that a sizable symmetric voltage appears in the naturally-oxidized-Cu/Ni_81_Fe_19_ bilayer, showing that the damping-like torque is generated in this system. By analyzing the ST-FMR spectral shape, we found that the damping-like torque efficiency is enhanced by more than an order of magnitude through natural oxidation of the Cu layer.

The observation of the enhancement of the spin-torque efficiency demonstrates that Cu becomes an efficient spin-torque generator through surface oxidation, despite the absence of heavy elements. This result shows that oxygen manipulation provides a way for efficient engineering of the spin-torque generator. To explore the possibility of oxidation-level engineering of the spin-orbit torques, we investigated the impact of the oxidation on the generation of the spin-orbit torque by Pt, the most widely used spintronic material. We found that the oxidation of Pt turns the heavy metal into an electrically insulating generator of the spin-orbit torques, which enables electrical switching of the perpendicular magnetization in a ferrimagnet sandwiched by insulating oxides.^31)^

**Figure 5.**
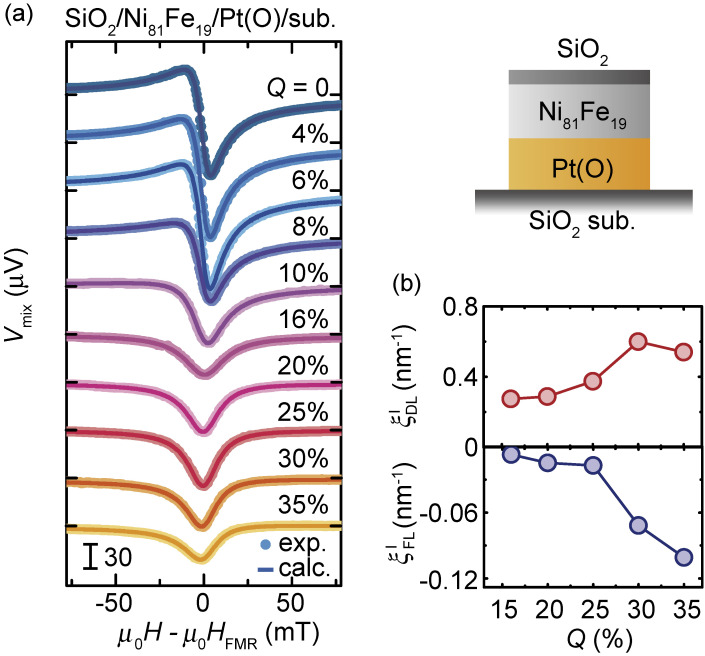
(Color online) (a) ST-FMR spectra for the SiO_2_/Ni_81_Fe_19_/Pt(O) devices at 7 GHz by changing *Q* from 0 to 35%. The solid circles are the experimental data and the solid curves are the fitting result. (b) *Q* dependence of the damping-like torque efficiency, $\xi_{\text{DL}}^{\text{I}}$ , and field-like torque efficiency, $\xi_{\text{FL}}^{\text{I}}$ . Figures adapted from Ref. 31.

To investigate the impact of oxidation on the spin-torque generation by Pt, we fabricated PtO_*x*_ films by reactive sputtering. For sputtering, argon and oxygen gases were introduced into the chamber and the amount of oxygen gas in the reactive mixture, *Q*, was varied. We measured the ST-FMR for Ni_81_Fe_19_/PtO_*x*_ bilayers, as shown in Fig. 5(a). Notably, the spectral shape of *V*_mix_ changes dramatically with *Q*. The ratio between the symmetric and antisymmetric components, *S*/*A*, increases with increasing *Q*, showing that the spin-orbit torques is strongly affected by the oxidation level of the Pt layer. In fact, the damping-like torque and field-like torque efficiencies are enhanced by increasing the oxidation level of the Pt layer, as shown in Fig. 5(b). By further increasing the oxidation level of Pt, we found that the spin-torque efficiency of the heavily-oxidized, electrically insulating PtO_*x*_ is nearly an order of magnitude larger than that of Pt.^79)^ The maximum SOT efficiency in this system reaches ξ_DL_ = 0.92, which corresponds to the damping-like-torque efficiency per applied electric field, $\xi_{\text{DL}}^{E}=8.7\times 10^{3}$ Ω^−1^ cm^−1^. The efficient generation of the spin-orbit torque by the insulating PtO_*x*_ suggests that the spin-orbit coupling at the Ni_81_Fe_19_/PtO_*x*_ interface is responsible for generating the spin-orbit torques.

**Figure 6.**
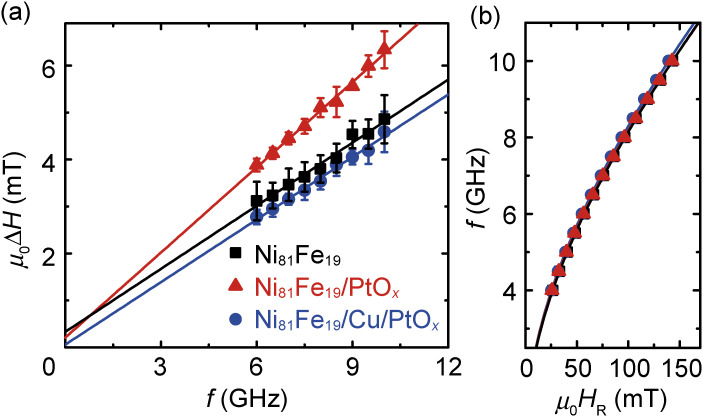
(Color online) (a) RF current frequency, *f*, dependence of the half-width at half-maximum μ_0_Δ*H* for the Ni_81_Fe_19_, Ni_81_Fe_19_/PtO_*x*_ and Ni_81_Fe_19_/Cu/PtO_*x*_ samples. The solid lines are the linear fit to the experimental data. (b) RF current frequency, *f*, dependence of the resonance field, μ_0_*H*_R_, for the three samples. The solid curves are the fitting result using the Kittel formula. Figures adapted from Ref. 98.

The strong spin-orbit coupling at the Ni_81_Fe_19_/PtO_*x*_ interface is evidenced by magnetic damping of the Ni_81_Fe_19_ layer.^98)^ The magnetic damping constant, α, can be quantified by fitting the RF current frequency *f* dependence of the FMR spectral width, μ_0_Δ*H*, using μ_0_Δ*H* = μ_0_Δ*H*_ext_ + (2πα/γ)*f*, where Δ*H*_ext_ and γ are the inhomogeneous linewidth broadening of the extrinsic contribution and the gyromagnetic ratio, respectively.^12,99)^ Figure 6(a) shows the *f* dependence of the FMR linewidth, μ_0_Δ*H*, for Ni_81_Fe_19_, Ni_81_Fe_19_/PtO_*x*_, and Ni_81_Fe_19_/Cu/PtO_*x*_ films. This result shows that the slope of the *f* dependence of μ_0_Δ*H* for the Ni_81_Fe_19_/PtO_*x*_ film is clearly larger than that for the Ni_81_Fe_19_ and Ni_81_Fe_19_/Cu/PtO_*x*_ films, demonstrating larger magnetic damping in the Ni_81_Fe_19_/PtO_*x*_ film. In contrast, the effective demagnetization field is the same in these devices as evidenced in Fig. 6(b). Here, the difference in α between the Ni_81_Fe_19_ and Ni_81_Fe_19_/Cu/PtO_*x*_ films is vanishingly small, which is within the experimental error. In contrast, the damping of the Ni_81_Fe_19_/PtO_*x*_ film is clearly larger than that of the other films, indicating that the direct contact between Ni_81_Fe_19_ and PtO_*x*_ is essential for enhancing the magnetization damping.

The enhanced magnetic damping can be attributed to spin pumping, which refers to the emission of a spin current from the ferromagnetic layer induced by the magnetization precession.^70)^ In ferromagnetic/nonmagnetic heterostructures, the spin current emitted from the ferromagnetic layer is absorbed in the nonmagnetic layer due to the bulk spin-orbit coupling and/or the ferromagnetic/nonmagnetic interface due to the interfacial spin-orbit coupling. Spin-current absorption deprives magnetization of the angular momentum, resulting in enhancing the magnetic damping.^70)^ Since the PtO_*x*_ layer is an insulator, the observed enhancement of the magnetic damping can be attributed to strong spin-orbit coupling at the Ni_81_Fe_19_/PtO_*x*_ interface.

**Figure 7.**
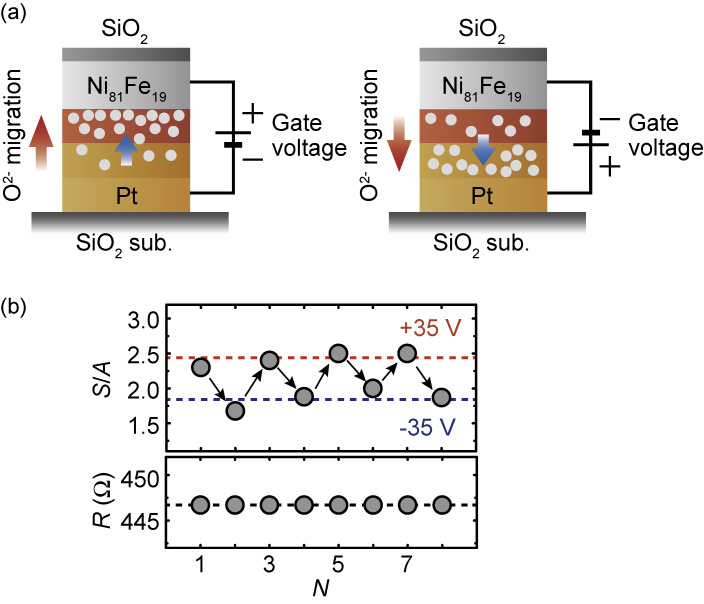
(Color online) (a) Schematic of the experimental setup for the application of the gate voltages used to drive the O^2−^ migration. The O^2−^ migrates towards the Ni_81_Fe_19_/PtO_*x*_ interface for the application of the positive gate voltage (left), whereas the negative gate voltage drives O^2−^ away from the Ni_81_Fe_19_/PtO_*x*_ interface (right). The gray solid circles represent oxygen ions. (b) The magnitude of the *S*/*A* ratio obtained by fitting the corresponding ST-FMR spectra, where *N* represents the cycle index. The ST-FMR measured after the application of the gate voltage of +35 V (*N* = 1, 3, 5, 7) or −35 V (*N* = 2, 4, 6, 8). The in-plane electrical resistance *R* of the Ni_81_Fe_19_ layer in the Ni_81_Fe_19_/PtO_*x*_/PtO_*y*_/Pt device measured after removing the applied voltages of ±35 V is plotted correspondingly. Figures adapted from Ref. 31.

The above results reveal the significant impact of Pt layer oxidation on interfacial spin-orbit coupling and spin-orbit torques in the Ni_81_Fe_19_/Pt structure. This result offers a way to tune the spin-orbit torques through voltage-driven O^2−^ migration near the Ni_81_Fe_19_/PtO_*x*_ interface. Voltage-driven O^2−^ migration has been well studied over a wide range of oxides,^100–106)^ and exploited as a mechanism for resistive switching in anionic metal/oxide/metal memristors.^100,101)^ A schematic illustration of the PtO_*x*_-based ST-FMR device is shown in Fig. 7(a). The platinum oxide layer is designed to be a PtO_*x*_ (oxygen deficient)/PtO_*y*_ (oxygen rich) structure with the top oxygen-deficient PtO_*x*_ serving as an oxygen-vacancy reservoir. This structure is reminiscent of oxide-based memristors.^105)^ When a positive gate voltage (0 V → +35 V → 0 V) was applied, the O^2−^ migrated towards the Ni_81_Fe_19_/PtO_*x*_ interface, leading to an increase of the *S*/*A* ratio due to high oxygen incorporation in the PtO_*x*_ layer (see Fig. 7(b)). In contrast, the application of a negative gate voltage (0 V → −35 V → 0 V), which drives O^2−^ away from the Ni_81_Fe_19_/PtO_*x*_ interface, results in a decrease of the *S*/*A* ratio. This is consistent with the oxidation level dependence of the ST-FMR spectral shape. Figure 7(b) demonstrates reversible switching of the *S*/*A* ratio induced by the voltage application. In spin-orbitronics, Pt has been one of the most efficient spin-torque source. This result shows that the spin-torque efficiency can be further enhanced by oxygen manipulation, promising a route for exploring efficient spin-torque generators through the oxidation of heavy metals.

## 6 Mechanism of interfacial spin-orbit torque

The mechanism of spin-orbit torque engineered by oxygen manipulation was investigated by using Ni_81_Fe_19_/CuO_*x*_ bilayers, where the great flexibility of the oxidation level of Cu provides a way to study the physics of the spin-orbit torques generated by interfacial spin-orbit coupling.^34)^ The devices used in this study were Ni_81_Fe_19_/CuO_*x*_ bilayers with various oxidation levels. The CuO_*x*_ layer was fabricated by reactive sputtering at various *Q* values. To quantify the spin-orbit torque arising purely from the interface, we measured the ST-FMR for Ni_81_Fe_19_/CuO_*x*_ bilayers with heavily oxidized, semi-insulating CuO_*x*_.

**Figure 8.**
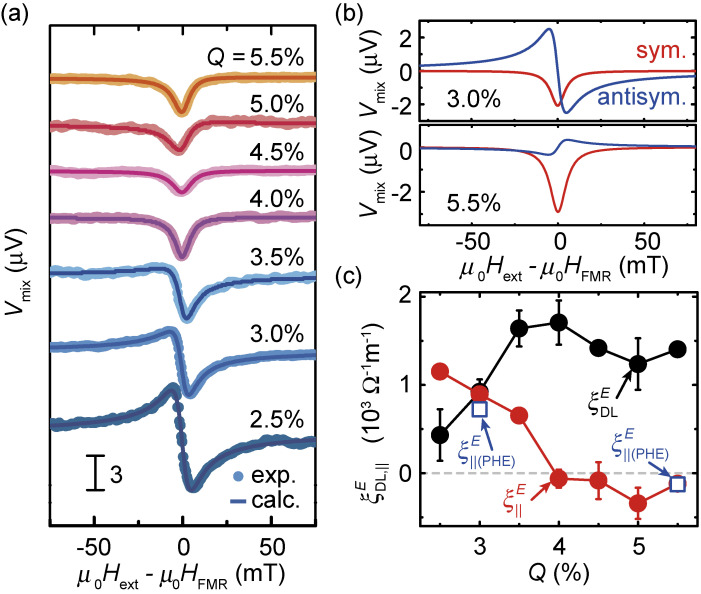
(Color online) (a) ST-FMR signal, *V*_mix_, as a function of the external field for the Ni_81_Fe_19_(7.5 nm)/CuO_*x*_(10 nm) bilayers with various *Q* measured at 7 GHz. (b) Fitting curves of *V*_mix_ as a function of the field for the Ni_81_Fe_19_(7.5 nm)/CuO_*x*_(10 nm) bilayers with *Q* = 3.0% and 5.5% at 7 GHz. The red and blue curves correspond to the symmetric and antisymmetric function fitting, respectively. (c) Estimated SOT efficiency per unit electric field, $\xi_{\text{DL}(∥)}^{E}$ , for the Ni_81_Fe_19_(7.5 nm)/CuO_*x*_(10 nm) bilayers with various *Q* values. The open squares are the in-plane torque efficiency, $\xi_{∥(\text{PHE})}^{E}$ , evaluated from second-harmonic Hall voltage measurements. The red and black solid circles are $\xi_{∥}^{E}$ and $\xi_{\text{DL}}^{E}$ , respectively, estimated from ST-FMR measurements. Figures adapted from Ref. 34.

Figure 8(a) shows the ST-FMR spectra for Ni_81_Fe_19_/CuO_*x*_ films with various *Q* values. This result shows that the ST-FMR signal appears in the present entire *Q* range, despite the fact that the current flow in the CuO_*x*_ layer is negligible, showing that the spin-orbit torques are generated by spin-orbit coupling at the Ni_81_Fe_19_/CuO_*x*_ interface. Figure 8(a) shows that the antisymmetric voltage is sensitive to the oxidation level of the CuO_*x*_ layer. Notable is that the sign of the antisymmetric voltage is reversed by changing *Q*, as shown in Fig. 8(b). The opposite sign of the antisymmetric component of the ST-FMR signals shows that the direction of the current-induced in-plane field is reversed by changing the oxidation level of the CuO_*x*_ layer. The sign reversal of the antisymmetric voltage indicates that the sign of the field-like torque is reversed by changing *Q* because the Oersted field due to the current flow in the CuO_*x*_ layer is negligible in Ni_81_Fe_19_/CuO_*x*_ film. Figure 8(c) shows the *Q* dependence of the damping-like and field-like torque efficiencies. This result shows that the damping-like torque is insensitive to the oxidation level of the CuO_*x*_ layer, while the field-like torque changes the sign by changing the oxidation level. The clear difference in the oxidation level dependence of the spin-orbit torques demonstrates that fundamentally different mechanisms are responsible for generating the damping-like and field-like spin-orbit torques in this system.

The Ni_81_Fe_19_/CuO_*x*_ bilayer can be approximately modeled as a two-dimensional Rashba ferromagnet where the conduction electrons’ spins are exchange-coupled to the magnetization. In this model, the damping-like and field-like spin-orbit torques are generated by two different mechanisms.^59)^ The damping-like torque originates from the Berry-phase curvature in the band structure^13)^; during the acceleration of carriers induced by an applied electric field, the spins tilt and generate a nonequilibrium out-of-plane spin polarization in response to an additional spin-orbit field, giving rise to the intrinsic damping-like torque. In contrast, the field-like torque in this model originates from the scattering of spin carriers at the Fermi surface. Since the extrinsic field-like torque has a conductivity-like behavior, it is sensitive to spin-dependent scattering, while the intrinsic damping-like torque is robust against disorders in the weak disorder regime. The observed behavior of the damping-like torque and the field-like torque is consistent with this scenario; the origin of the observed sign change of the field-like torque can be attributed to the variation of the spin-dependent disorder scattering at the interface.

**Figure 9.**
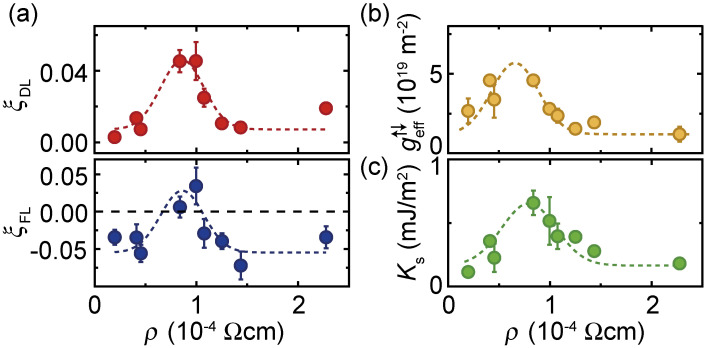
(Color online) (a) Damping-like ξ_DL_ and field-like ξ_FL_ spin-torque generation efficiencies for Ni_81_Fe_19_/CuO_*x*_ bilayers with 0.25% ≤ *Q* ≤ 1.25% as a function of the CuO_*x*_-layer resistivity, ρ. The solid circles are the experimental data and the dotted curve is a guide for eyes. (b) Effective spin mixing conductance, $g_{\text{eff}}^{↑↓}$ , for the Ni_81_Fe_19_/CuO_*x*_ bilayers. (c) Interface PMA energy density *K*_s_ for the Ni_81_Fe_19_/CuO_*x*_ bilayers. Figures adapted from Ref. 32.

The spin-orbit torques arising from the ferromagnetic-metal/metal-oxide interface can be maximized by a fine-tuning of the interfacial oxidation level.^32)^ Figure 9(a) shows damping-like ξ_DL_ and field-like ξ_FL_ spin-torque generation efficiencies for Ni_81_Fe_19_/CuO_*x*_ bilayers with 0.25% ≤ *Q* ≤ 1.25% as a function of the CuO_*x*_-layer resistivity, ρ. Here, the electrical resistivity, ρ, characterizes the oxidation level of the CuO_*x*_ layer because ρ is quite sensitive to the oxidation level, and increases monotonically with the oxidation level. Notable is that ξ_DL_ is significantly enhanced, whereas the sign of ξ_FL_ is reversed, only within a narrow range of the oxidation level of CuO_*x*_.

The dramatic change of the spin-torque efficiencies originates from an enhancement of the interfacial spin-orbit torques, which are maximized only within the narrow range of the oxidation level. In the moderately oxidized CuO_*x*_ (*Q* ≤ 1.25%), the applied change current can flow in the CuO_*x*_ layer, and thus both bulk and interface spin-orbit coupling can contribute to the spin-orbit torques. We found that the anomaly in the spin-orbit torques disappears upon inserting an ultrathin Cu layer between the Ni_81_Fe_19_ and CuO_*x*_. This result indicates that the enhancement of the damping-like torque and the sign reversal of the field-like torque arise from an enhancement of the interfacial spin-orbit torques; the interface spin-orbit torque efficiency is dramatically enhanced by oxidation only at ρ ∼ 9 × 10^−5^ Ω cm, and the dominant mechanism of the spin-torque generation is changed at only around this oxidation level.

Maximization of the interfacial spin-orbit torques is associated with enhancing the effective spin-mixing conductance, $g_{\text{eff}}^{↑↓}$ , and the interface perpendicular magnetic anisotropy (PMA) energy density, *K*_s_, as shown in Figs. 9(b) and 9(c). In the Ni_81_Fe_19_/CuO_*x*_ bilayer, $g_{\text{eff}}^{↑↓}$ can be attributed to an enhancement of the spin memory loss due to interface interfacial spin-orbit coupling.^107)^ We note that the enhancement of *K*_s_ by fine-tuning the interfacial oxidation level is consistent with previous reports.^39)^ The interface PMA arises from spin-orbit coupling in combination with orbital hybridization at the interface, which is quite sensitive to the interface oxidation level.^39)^ The observed concomitant enhancement of the interfacial spin-orbit torque and interfacial PMA indicates the important role of the orbital hybridization in generating interfacial spin-orbit torques.

The critical enhancement of the spin-orbit torque originating from the Ni_81_Fe_19_/CuO_*x*_ interface can be attributed to oxygen-induced deformations of the interface-state wave functions. The physics behind the interface spin-orbit coupling is orbital hybridization due to the broken inversion symmetry. The orbital hybridization due to the broken inversion symmetry deforms the interface-state wave function. *Ab initio* calculations show that the strength of the interface spin-orbit coupling is determined by the asymmetry of the interface-state wave function, or the strength of the hybridization, near to the position of the nucleus of the interface atoms, in conjunction with the atomic spin-orbit coupling.^108–115)^ Although the atomic spin-orbit coupling of O is quite weak, the incorporation of O atoms can dramatically modify the hybridization of the Ni, Fe, and Cu orbitals near to the interface. This oxygen-induced wave-function deformation can result in an enhancement of the interfacial spin-orbit coupling, or the interfacial spin-orbit torque. Maximization of the interfacial spin-orbit torque illustrates the essential role of the atomic scale and chemical bonding effects in the interface spin-orbit physics, providing a way to tune the spin-orbit torques by atomic modification.

The above studies focus on the impact of oxidation of the nonmagnetic side on generating spin-orbit torques in ferromagnetic-metal/nonmagnet structures. It is natural to expect that oxidizing the ferromagnetic side near to the interface also alters spin-orbit torques. We have investigated the role of oxidizing the ferromagnetic layer in the generation of spin-orbit torques, and found distinct differences in the interfacial oxidation effect in Pt/Ni_81_Fe_19_ and Pt/Co bilayers.^116)^ The interfacial oxidation of the ferromagnetic side in the Pt/Ni_81_Fe_19_ film suppresses the damping-like torque, while it reverses the direction of the field-like torque. In contrast, in Pt/Co film, oxidation of the Co layer near the interface enhances both damping-like and field-like torques.

We found that interfacial oxidation enhances the interfacial spin-orbit coupling in the Pt/Co film, and the dominant source of the spin-orbit torques in the Pt/CoO_*x*_/Co film is the spin-orbit coupling at the Pt/CoO_*x*_ interface. In contrast, interfacial spin-orbit coupling plays a minor role in Pt/Ni_81_Fe_19_ film, even when the Ni_81_Fe_19_ layer near the interface is oxidized. In this system, the bulk spin-orbit coupling is responsible for the spin-orbit torques, and the oxidation-induced change of the spin-orbit torques can be attributed to the change of the real Re[*G*^↑↓^] and imaginary Im[*G*^↑↓^] parts of the spin mixing conductance; the interfacial oxidation suppresses Re[*G*^↑↓^], while it reverses the sign of Im[*G*^↑↓^]. These results demonstrate that interfacial oxidation provides an effective way to manipulate the strength and sign of the spin-orbit torques.

## 7 Spin-orbit torque manipulation by hydrogen

**Figure 10.**
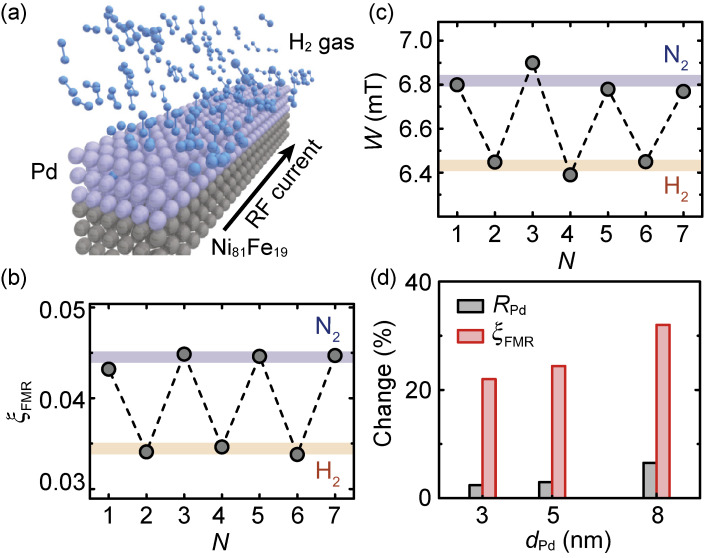
(Color online) (a) Schematic of the setup for the ST-FMR under the application of N_2_ and H_2_ gases for the Pd/Ni_81_Fe_19_ device. (b) Spin-torque efficiency, ξ_FMR_, and (c) ST-FMR linewidth, *W*, measured by alternatively applying N_2_ and H_2_ gases. *N* represents the cycle index. (d) Comparison of the change in the resistance, *R*_Pd_, of the Pd layer and the spin-torque efficiency, ξ_FMR_, for the Pd/Ni_81_Fe_19_ films with different Pd-layer-thicknesses (*d*_Pd_) induced by applying N_2_ and H_2_ gases. Figures adapted from Ref. 117.

Manipulating the spin-orbit torque can be achieved by using hydrogen, as well as oxygen. We found that the spin-orbit torque generated by Pd can be reversibly manipulated by the absorption and desorption of H_2_ gas.^117)^ Here, Pd is well known as an efficient H_2_ absorber. The resistance of Pd is known to be manipulated by the absorption and desorption of H_2_ gas. The resistance change has been widely studied for applications as hydrogen sensors.^118–120)^ Figure 10(a) shows a schematic illustration of a Pd/Ni_81_Fe_19_ device used for investigating spin-torque manipulation using H_2_ gas. By measuring the ST-FMR for this device, we found that the FMR spin-torque efficiency, ξ_FMR_, is reversibly manipulated by applying N_2_ or H_2_ gas, as shown in Fig. 10(b). Since the spin-orbit torque is dominated by the bulk spin Hall effect in the Pd layer, and the field-like torque is negligible in the Pd/Ni_81_Fe_19_ structure, ξ_FMR_ corresponds to *T*_int_θ_SHE_, where *T*_int_ is the interfacial spin transparency and θ_SHE_ is the spin Hall angle, the ratio between the spin Hall conductivity and the electric resistivity of Pd.^121)^ This indicates that the observed change of ξ_FMR_ is caused by the change of *T*_int_ and/or θ_SHE_ due to the absorption and desorption of H_2_ gas. Here, the difference of the H_2_ concentration between the environment and the Pd film is the driving force for the H_2_ absorption and desorption process^122)^; by applying H_2_ gas, the small H atom can easily dissolve into the Pd film and form metastable PdH_*x*_, while in the case of applying N_2_, the H atoms in the Pd film diffuse into the environment and form H_2_.

The origin of the reversible manipulation of the spin-orbit torque can be attributed to the reversible change of the spin diffusion length in the Pd layer induced by the absorption and desorption of H_2_ gas. As shown in Fig. 10(c), the ST-FMR spectral width, *W*, is also changed by the absorption and desorption of H_2_ gas, indicating that the effective spin-mixing conductance, $g_{\text{eff}}^{↑↓}$ , is reversibly manipulated. The change of the effective spin-mixing conductance indicates the change of the spin diffusion length of the Pd layer.^70)^ The change of the spin diffusion length can also change the spin transparency, *T*_int_, because *T*_int_ is set by the spin backflow. We found that the reversible manipulation of ξ_FMR_ and $g_{\text{eff}}^{↑↓}$ can be consistently reproduced by assuming that the spin diffusion length of the Pd layer is changed from 2 nm to 3.5 nm by H_2_ absorption. This suggests that the change of the spin-orbit torque is dominated by the change of the interfacial spin transparency, rather than the possible change of θ_SHE_.

Our experimental finding is that the spin-orbit torque generated by Pd can be reversibly manipulated by the H_2_ absorption and desorption. The change of the spin-torque efficiency is almost an order of magnitude larger than that of the resistance, as shown in Fig. 10(d). This result not only provides a novel approach to manipulate the spin-orbit torque, but also paves a way to apply spin-orbitronic technology in a variety of fields.

## 8 Molecular engineering of spin-orbit torques

The molecular engineering of spin-orbitronic devices using self-assembled organic monolayers (SAMs) provides a way to control the spin-charge conversion and spin-orbit torques. SAMs offer a powerful way to alter the properties of solid-state surfaces,^123–128)^ providing diverse applications, such as corrosion inhibition, nanopatterning, sensors, and molecular electronic devices.^127,128)^ In electronics, SAMs have been widely used to tune the electronic properties of oxides, semiconductors, and metals.^123–126)^ The modification of magnetic and spintronic properties has also been investigated in the field of spinterface.^129,130)^ We have demonstrated that the decoration of SAMs on Bi/Ag/CoFeB trilayers enables one to change the strength of the Rashba-Edelstein effect at the Bi/Ag interface.^15)^

**Figure 11.**
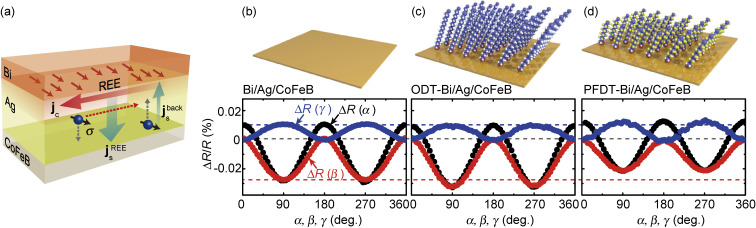
(Color online) (a) Schematic illustration of REMR in a Bi/Ag/CoFeB trilayer induced by the Rashba-Edelstein effect at the Bi/Ag interface. The change in the longitudinal resistance, Δ*R*, of (b) the Bi(5 nm)/Ag(2 nm)/CoFeB(2.5 nm) trilayer, (c) ODT-Bi(5 nm)/Ag(2 nm)/CoFeB(2.5 nm) trilayer, and (d) PFDT-Bi(5 nm)/Ag(2 nm)/CoFeB(2.5 nm) trilayer, as a function of the rotation of the magnetic field of 6 T, where *R* is the longitudinal resistance at μ_0_*H* = 0. Schematic illustrations of the pristine Bi/Ag/CoFeB trilayer and SAM-decorated Bi/Ag/CoFeB trilayers are also shown. Figures adapted from Ref. 15.

Molecular engineering of spin-charge conversion was evidenced by measuring the Rashba-Edelstein magnetoresistance (REMR).^131)^ Figure 11(a) shows a schematic illustration of the REMR. In the Bi/Ag/CoFeB trilayer, the Rashba-Edelstein effect at the Bi/Ag interface generates a diffusive spin current in the Ag layer. This spin current is reflected at the Ag/CoFeB interface, and then converted into a charge current through the inverse Rashba-Edelstein effect at the Bi/Ag interface. Since the reflection of the spin current depends on the relative orientation of the magnetization and the spin-polarization direction of the spin current, the additional charge current arising from this process depends on the magnetization direction. Thus, the device resistance depends on the magnetization direction due to the Rashba-Edelstein effect and spin-current reflection, which is the mechanism of the REMR. Figure 11(b) shows the change in the longitudinal resistance, Δ*R*, of the Bi/Ag/CoFeB trilayer during the rotation of an applied magnetic field, μ_0_*H* = 6 T, where α, β, and γ are the rotation angles.^15)^ Of particular importance is Δ*R*(β). Here, the REMR can be expressed as Δ*R*(β) ∼ −sin^2^ β. The observed Δ*R*(β) result shown in Fig. 11(a) is consistent with this scenario, showing that the REMR dominates the Δ*R*(β) in the Bi/Ag/CoFeB trilayer.

Our finding is that the strength of the REMR can be tuned by the molecular self-assembly on the Rashba spin-orbit device. Figures 11(c) and 11(d) show Δ*R* of the Bi/Ag/CoFeB trilayer decorated with 1-octadecanethiol (ODT) and 1*H*,1*H*,2*H*,2*H*-perfluorodecanethiol (PFDT), respectively. This result demonstrates that Δ*R*(β) is enhanced by the ODT decoration, while Δ*R*(β) is suppressed by the PFDT decoration. Here, we note that Δ*R*(γ) is not affected by the SAM formations. The negligible change in Δ*R*(γ) indicates that the magneto-electric property of the CoFeB layer is not altered by the SAM formations, since Δ*R*(γ) ∼ sin^2^ γ originates from the anisotropic magnetoresistance of the CoFeB layer. This shows that the observed change in Δ*R*(β) arises from the change of the Rashba-Edelstein effect at the Bi/Ag interface, induced by the molecular self-assembly.

**Figure 12.**
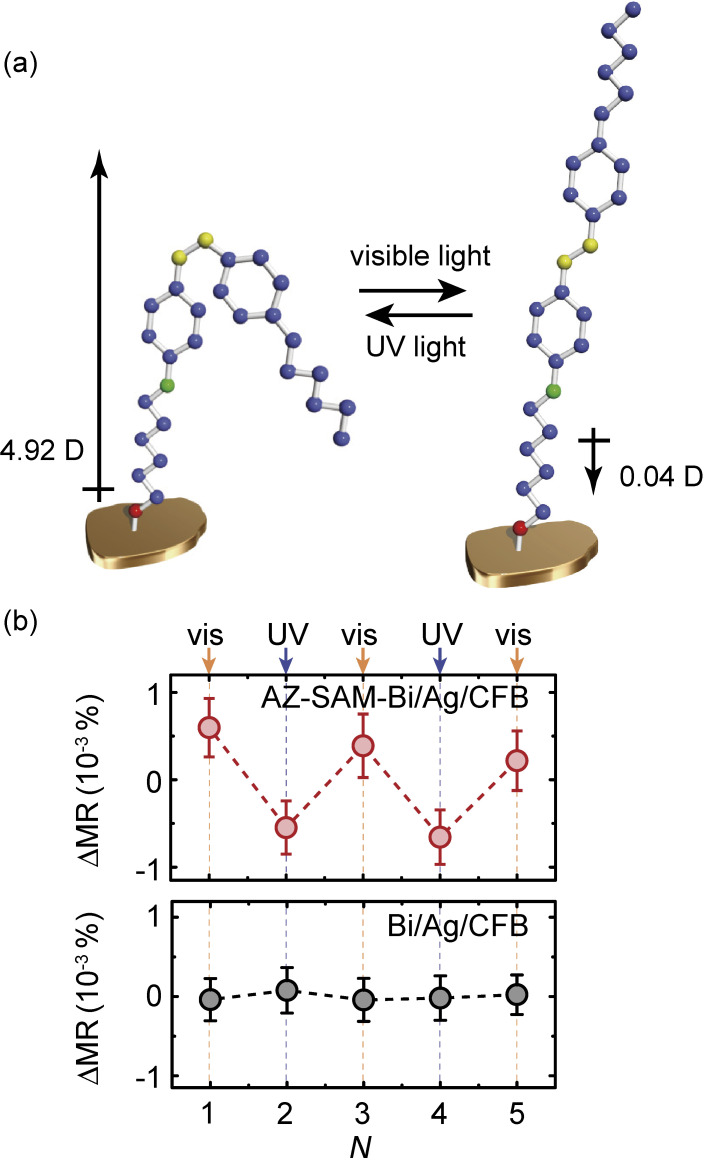
(Color online) (a) Illustration of the reversible *cis*-*trans* photoisomerization of AZ-SAM formed on the Bi/Ag/CoFeB trilayer under UV and visible light irradiation. The arrows represent the dipole moment of AZ obtained from density functional theory calculations. (b) Change in the REMR ratio, ΔMR = MR − MR_avg_, for the AZ-SAM-decorated Bi/Ag/CoFeB trilayer and the pristine Bi/Ag/CoFeB trilayer measured after the visible (*N* = 1, 3, 5) or UV (*N* = 2, 4) light irradiation. Here, MR ≡ [Δ*R*(β = 0) − Δ*R*(β = 90°)]/*R*, where *N* represents the cycle index. MR_avg_ is the average MR for *N* = 1, 2, 3, 4, 5. Figures adapted from Ref. 15.

The advantage of the molecular engineering of spintronic devices is that further functionalities can be incorporated into the spin-orbit device by utilizing functional molecules. We have demonstrated reversible phototuning of the Rashba-Edelstein effect through light-driven molecular transformations using an azobenzene-functionalized SAM, which can reversibly isomerize between *trans* and *cis* forms under photo-irradiation.^15)^ To induce molecular transformation of the AZ-SAM, the AZ-SAM-decorated Bi/Ag/CoFeB trilayer was irradiated with ultraviolet (UV) light or visible light for three minutes (see Fig. 12(a)). After irradiation, we measured the magnetoresistance without irradiation. As shown in Fig. 12(b), the REMR of Bi/Ag/CoFeB decorated with AZ-SAM is reversibly manipulated by the visible (*N* = 1, 3, 5) and UV (*N* = 2, 4) light irradiation, where *N* represents the cycle index. This result demonstrates the reversible tuning of the Rashba-Edelstein effect by light-driven molecular transformation. This interpretation is supported by the fact that the REMR ratio is not affected by the UV and visible light irradiation in the pristine Bi/Ag/CoFeB trilayer, where the AZ-SAM is absent, as shown in Fig. 12(b).

**Figure 13.**
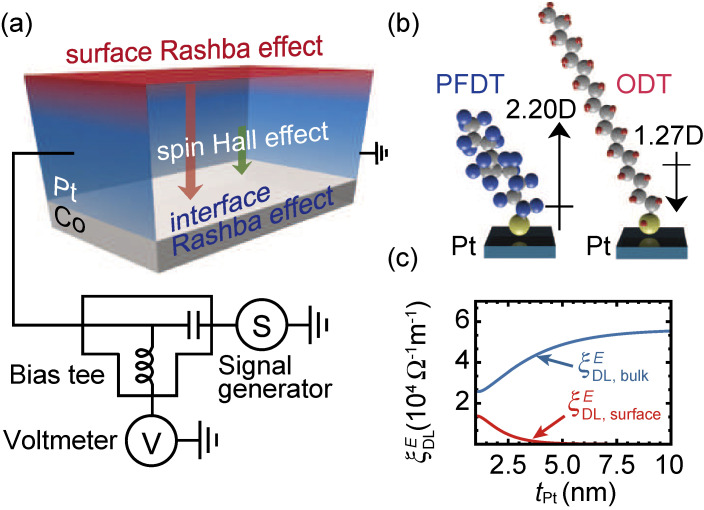
(Color online) (a) Schematic illustration of the Pt/Co bilayer and experimental setup. (b) Schematic illustration of ODT and PFDT molecules on the Pt surface. The arrows represent the dipole moment of the SAM-forming molecules. (c) Pt-layer thickness, *t*_Pt_, dependence of $\xi_{\text{DL},\text{surface}}^{E}$ and $\xi_{\text{DL},\text{bulk}}^{E}$ for Pt/Co films. Figures adapted from Ref. 132.

The molecular tuning of solid-state surfaces provides a way to control the spin-orbit torque arising from the surface Rashba-Edelstein effect. Manipulation of the spin-orbit torque originating from the surface Rashba-Edelstein effect has been demonstrated by measuring the spin-orbit torques generated by an ultrathin Pt, decorated by SAMs.^132)^ The effect of the SAM formation on the spin-orbit torques was studied in Pt/Co films, as shown in Fig. 13(a). The surface of the 1-nm-thick Pt layer was decorated with ODT or PFDT (see Fig. 13(b)). The ST-FMR for the SAM-decorated Pt/Co films demonstrates that the damping-like torque is enhanced by PFDT formation, while it is suppressed by ODT formation. The field-like torque is unaffected by the SAM formation. In the Pt/Co bilayer, the spin-orbit torques can be generated by the spin Hall effect in the Pt layer, the Rashba-Edelstein effect at the Pt/Co interface, and the Rashba-Edelstein effect at the Pt surface, as shown in Fig. 13(a). The spin Hall effect and the surface Rashba-Edelstein effect generate spin-orbit torques through the spin-transfer mechanism, which primarily generates a damping-like torque. In contrast, the interface Rashba-Edelstein effect primarily exerts a field-like torque on the magnetization through interfacial exchange coupling. The negligible change in the field-like torque indicates that the interface Rashba-Edelstein effect is unaffected by the SAM decoration, which is reasonable because the charge screening length in the Pt layer is much shorter than the thickness.

Molecular tuning of the damping-like torque indicates that the surface Rashba-Edelstein generates a sizable spin-orbit torque in the ultrathin-Pt/Co bilayers. In the Pt/Co bilayer, the change of the bulk spin Hall effect, as well as the interface Rashba-Edelstein effect, due to molecular self-assembly can be assumed to be negligible, which is supported by the fact that the resistance of the bilayer is almost unchanged by the SAM decoration. From the measured values of the damping-like torque efficiency, the damping-like torque efficiency due to the bulk spin Hall effect, $\xi_{\text{DL},\text{bulk}}^{E}$ , and surface Rashba-Edelstein effect, $\xi_{\text{DL},\text{surface}}^{E}$ , can be calculated by using the standard drift-diffusion model, as shown in Fig. 13(c). From this result, we found that the PFDT formation enhances the surface Rashba-Edelstein spin-orbit torque by 39%, while the ODT formation suppresses it by 27%. The molecular tuning of the surface Rashba-Edelstein effect is consistent with density functional theory calculations, which show that the out-of-plane buckling of Pt atoms plays an important role in enhancing the surface Rashba-Edelstein spin-orbit torque induced by PFDT formation on the Pt surface. These results illustrate the crucial role of the surface spin-orbit coupling in generating the spin-orbit torque. This finding provides an essential information for the fundamental understanding of the spin-orbit torques, since the spin-orbit torques have generally been attributed to two mechanisms: the interface Rashba-Edelstein effect and bulk spin Hall effect.^8)^

## 9 Electric control of spin-orbit torques

The electric modulation of the spin-orbit torques in metallic spin-orbitronic structures has generally been limited to be only below 10%.^133)^ We have shown that an ionic-liquid gating of ultrathin Au enables effective control of the spin-orbit torque in the conventional oxide/heavy-metal/ferromagnetic-metal heterostructure.^134)^

**Figure 14.**
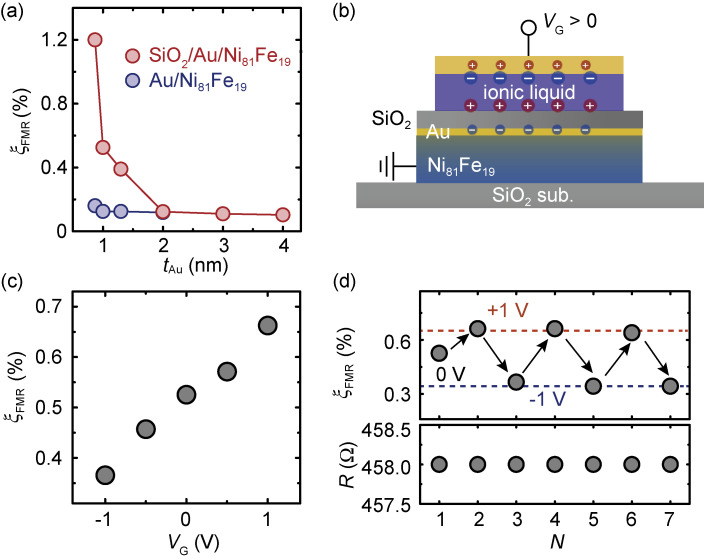
(Color online) (a) Dependence of the spin-torque generation efficiency, ξ_FMR_, on the Au thickness, *t*_Au_, for Au/Ni_81_Fe_19_ and SiO_2_/Au/Ni_81_Fe_19_ devices, respectively. (b) Schematic of the cross-section view along the junction by applying a positive gate voltage of *V*_G_ > 0. (c) Dependence of the spin-torque generation efficiency, ξ_FMR_, on the applied gate voltages, *V*_G_. (d) ξ_FMR_ as a function of the reversible gate voltages, *V*_G_ = ±1 V. The corresponding in-plane resistance, *R*, as a function of the applied gate voltages is also shown. *N* represents the circle index. Figures adapted from Ref. 134.

As shown in Fig. 14(a), we found that by simply depositing a SiO_2_ capping layer on an ultrathin-Au/Ni_81_Fe_19_ film, the spin-torque efficiency, ξ_FMR_, is enhanced by a maximum of seven times. We also found that the efficient spin-torque generation survives even when inserting an ultrathin Ti layer between SiO_2_ and Au layers. This result shows that the spin-orbit torque in the SiO_2_/Au/Ni_81_Fe_19_ film cannot be attributed to the Rashba effect at the SiO_2_/Au interface. The negligible role of the Rashba effect in the generation of a spin-orbit torque indicates that the enhancement of the spin-torque efficiency induced by depositing a SiO_2_ capping layer can be attributed to modified interface spin-orbit scattering at the rougher interface in the case of ultrathin Au.^135)^

Spin-torque generation by ultrathin Au can be manipulated by ionic-liquid gating. Figure 14(b) shows the device structure used for the gate control of the spin-orbit torque. As shown in Fig. 14(c), by applying gate voltages, *V*_G_, with different magnitudes and signs, the spin-torque efficiency, ξ_FMR_, decreases when applying negative gate voltages, while it increases when applying positive gate voltages. This result shows that ξ_FMR_ changes from 0.36% to 0.66% by changing the gate voltage from −1 V to 1 V. Furthermore, ξ_FMR_ switches reversibly upon reversing the polarity of the gate voltage, as shown in Fig. 14(d), while the change of the resistance is negligible. This result demonstrates a reversible control of the spin-orbit torque efficiency by a factor of two with only a gate voltage of ±1 V. Demonstrating the electric control of the spin-orbit torque can potentially add new functionalities to spin-orbit devices, such as simultaneous memory and logic functions.

## 10 Conclusions

We have reviewed our studies concerning the generation and manipulation of current-induced spin-orbit torques. The physics behind the current-induced spin-orbit torques is the conversion between a charge current into a spin current and/or spin polarization. The spin-charge conversion has played a crucial role for establishing spin-dependent phenomena; the conversion from charge to spin, triggered by the spin-orbit coupling, enables electric manipulation of the magnetization through spin-orbit torques, while the inverse process, a conversion from spin to charge, allows electric detection of spin currents. The charge-spin conversion originating from spin-orbit coupling provides a way to reveal a variety of phenomena arising from spin currents.

Whereas the basic mechanisms behind spin-orbit torques are understood, owing to studies in this field over the past decade, a robust and systematic quantitative agreement between theory and experiment is still lacking. An example is the charge-spin conversion efficiency and interfacial spin transparency in metallic devices; reported values vary even for the same system. The quantification of these parameters requires knowledge about spin mixing conductance, as well as spin-orbit torques. Although the spin mixing conductance is typically determined by measuring the magnetic damping, recent studies have suggested that extraction of the parameter requires a careful determination of the two-magnon scattering, as well as the interface spin memory loss, which has previously been neglected.^136,137)^ This suggests that explorating the generation and manipulation of spin-orbit torques with further careful experiments will provide essential information for a fundamental understanding of spin-orbit physics in solid-state devices.

Spin-orbit torques have proved to be an exciting new opportunity for efficient electrical control of magnetization states. Spin-orbit torques enable not only to switch magnetization, but also to drive the motion of magnetic textures, such as domain walls and skyrmions. Another attractive feature is their ability to excite any type of magnetic materials, ranging from metals to semiconductors and insulators, in both ferromagnetic and antiferromagnetic states. This versatility of the spin-orbit torques promises a way to realize a plethora of ultralow power and fast spintronic devices, such as nonvolatile magnetic memories, nanoscale microwave/terahertz sources, and neuromorphic computing devices.
